# BioCreative VI Precision Medicine Track system performance is constrained by entity recognition and variations in corpus characteristics

**DOI:** 10.1093/database/bay122

**Published:** 2018-12-14

**Authors:** Qingyu Chen, Nagesh C Panyam, Aparna Elangovan, Karin Verspoor

**Affiliations:** School of Computing and Information Systems, The University of Melbourne, Parkville VIC Australia

## Abstract

Precision medicine aims to provide personalized treatments based on individual patient profiles. One critical step towards precision medicine is leveraging knowledge derived from biomedical publications—a tremendous literature resource presenting the latest scientific discoveries on genes, mutations and diseases. Biomedical natural language processing (BioNLP) plays a vital role in supporting automation of this process. BioCreative VI Track 4 brings community effort to the task of automatically identifying and extracting protein–protein interactions (PPi) affected by mutations (PPIm), important in the precision medicine context for capturing individual genotype variation related to disease.

We present the READ-BioMed team’s approach to identifying PPIm-related publications and to extracting specific PPIm information from those publications in the context of the BioCreative VI PPIm track. We observe that current BioNLP tools are insufficient to recognise entities for these two tasks; the best existing mutation recognition tool achieves only 55% recall in the document triage training set, while relation extraction performance is limited by the low recall performance of gene entity recognition. We develop the models accordingly: for document triage, we develop term lists capturing interactions and mutations to complement BioNLP tools, and select effective features via a feature contribution study, whereas an ensemble of BioNLP tools is employed for relation extraction.

Our best document triage model achieves an F-score of 66.77% while our best model for relation extraction achieved an F-score of 35.09% over the final (updated post-task) test set. Impacting the document triage task, the characteristics of mutations are statistically different in the training and testing sets. While a vital new direction for biomedical text mining research, this early attempt to tackle the problem of identifying genetic variation of substantial biological significance highlights the importance of representative training data and the cascading impact of tool limitations in a modular system.

## Introduction

Precision medicine is an emerging field (1), aiming to provide specialized medical treatments on the basis of individual patient characteristics, including their genotype, phenotype and other diagnostics (2). Primary biomedical databases represent an extraordinary collective volume of work, comprised of millions of contributions from the biomedical research community over decades (3). For instance, PubMed, the primary biomedical literature database, contains over 28 million biomedical publications (https://www.ncbi.nlm.nih.gov/pubmed/). This literature represents a critical information source for precision medicine, but the vast quantities of unstructured text make it challenging to identify and navigate relevant evidence. Biomedical Natural Language Processing (BioNLP) can be applied to address this problem, with the aim of automatically transforming publications into structured, searchable data. Two primary BioNLP tasks relevant to precision medicine are named entity recognition, e.g. as applied to recognize mentions of mutations in articles (4) and relation extraction, e.g. to identify interactions, such as protein–protein interactions (PPI), between biological entities described in papers (5). Few attempts have been made to closely integrate these tasks to understand higher-level interactions between them; recent efforts such as LitVar (6) emphasize sentence-level co-occurrence of entities but do not consider higher-order interactions between entities and relations. In the context of precision medicine, identification and extraction of PPI affected by mutations (PPIm) described in the literature (8) supports synthesis and, in turn, deeper understanding of the biological impacts of genetic variation.

The BioCreative VI Track 4 aimed to bring community effort to tackle this particular challenge (7). It established a gold standard dataset, consisting of 5509 biomedical articles that were manually annotated for PPIm statements (7). The track offered two related tasks for participation: (i) document triage, classifying whether or not a document is relevant to PPIm and returning the top-ranked relevant documents; and (ii) relation extraction, examining the document to identify specific protein pairs whose interaction is affected by a mutation. These tasks aim to support curation of the information relevant to precision medicine such that effort by human biocurators to catalogue PPIm facts is more effective; biocurators can focus on the top-ranked relevant documents without tedious manual examination of large quantities of irrelevant documents. This in turn facilitates precision medicine.

An example of a relevant PPIm relation that is expected to be identified in the relation extraction task appears in the sentence *`LAF1*, an R2R3-MYB factor, interacts with *HFR1*, a basic helix–loop–helix (bHLH) factor, and this interaction is abolished by the *R97A* mutation in the LAF1 R2R3 domain.’ [PMID:17699755], where the `LAF1–HFR1*’* interaction is impacted by the *R97A* mutation in *LAF1*.

In the official results of this shared task presented at the BioCreative VI workshop, our best document triage model achieved over 88% recall, achieving the third highest recall amongst 22 submissions, while our best model for relation extraction achieved a Micro F1-score of 37.17%, ranking second amongst six submissions just behind the top team at 37.29%. Updated results provided post-workshop after correction of the test set lift our system to top rank across three out of four measures (described in detail below).

In this work, we present the READ-Biomed team system developed for BioCreative VI Track 4, for detection of documents relevant to PPIm and extraction of PPIm relations, substantially extending the original system description paper (9). Specifically, there are three main additional contributions beyond our original submission.

- We provide an in-depth investigation of the original training set, by quantifying the effectiveness of a range of standard BioNLP tools for biological entity recognition. Application of these tools is a typical first important step in biocuration workflows and text mining pipelines. The analysis shows that existing BioNLP tools alone are not sufficient for recognising mutations or interactions. For example, the best mutation recognizer can achieve only ∼55% recall in the document triage data set; similarly, the maximum recall performance achievable by any relation extraction system is 56% on the provided data set when only using the standardly available tools. This suggests that the proposed models should leverage other techniques beyond standard BioNLP tools. It also shows that achieving high recall, especially for document triage task, is essential: low recall of relevant documents has propagated impacts on the later relation extraction step.
- We study our models more comprehensively than in the original submission. This includes a detailed description of the feature engineering; providing a feature contribution study to quantify the effects of different sets of features in both tasks. For document triage, we leverage BioNLP tools and term lists to better capture mutations and interactions and experiment with several classification algorithms to detect relevant documents. The best model achieves ∼89% recall, significantly overcoming the limitations of the current BioNLP tools. For relation extraction, we study machine learning methods using term lists and dependency graph kernels and a heuristically designed co-occurrence based approach. Coupled with standard BioNLP tools for entity recognition, we achieve an F-score of 35.09 and 34.92% on the test using the machine learning and co-occurrence based approaches, respectively.
- We provide an in-depth error analysis of our performance on both focus tasks, and a case study for a document triage model. The results collectively show that mutation characteristics have different impacts on the two tasks. For document triage, the model performance is dramatically decreased due to statistically significant differences in mutation characteristics between the training and testing set.

## Analysis of the training set

Much work in BioNLP applies supervised machine learning to learn task models. Building supervised learning models, in general, consists of examining the characteristics of the training sets, developing features accordingly, choosing appropriate models, training and validating these models via standard evaluation metrics, e.g. precision and recall for classification tasks, and ultimately applying the developed models to the testing sets. Underlying this approach, there are two critical and implicit assumptions: (i) the characteristics of the testing set are similar to the provided training set (i.e. derived from similar distributions), such that the models capturing the important features and characteristics from the training set can be applied to the testing set and (ii) while the characteristics are similar, the testing set instances are novel; they are ‘hidden’ during the training process, and the evaluation over the testing set is a test of the generalisation of the trained model to unseen instances. Under these assumptions, analysis of the characteristics of the training set is the first step in developing supervised models. In this section, we investigate the BioCreative VI Track 4 training set and illustrate how it relates to the two focus tasks.

### PPIm document triage

The document triage task is framed as a binary text classification task, i.e. classifying whether a document is relevant for PPIm or not. However, the task is related to a targeted information retrieval task, where the objective is to output documents ranked in order of relevance. The notion of relevance is context-dependent (10); a document in the context of this task is relevant if it describes PPI impacted by mutations. In other words, a relevant document must (i) mention at least one mutation, (ii) describe at least one protein interaction and (iii) indicate that there is some change in the interaction that can be considered to be caused by the mutation. To classify a document, standard text classification methods make use of tokens in the text; in the context of the PPIm task, terms corresponding to mutations, proteins and interactions may be pre-identified using entity recognition tools and given special status. A number of popular BioNLP entity recognition tools have been widely used for such entities; for instance, tmVar (4, 11), GNormPlus (12) and PIE the search (13) can be used to identify mutations, genes/proteins and possible interactions, respectively. PIE the search outputs the probability that a document contains an interaction, whereas tmVar and GNormPlus outputs individual recognized entities.

Thus, as the first step to examining the characteristics of the training set, we apply existing BioNLP entity recognition tools to recognize mutations and interactions. We quantify (i) how different the relevant and non-relevant documents are in terms of entities, e.g. if there are many more mutations identified in the relevant instances, it may be an important feature to distinguish the two classes and (ii) how well the tools identify mutations and interactions in the PPIm-relevant instances in the training set. Given the task definition, relevant documents would be expected to mention at least one mutation and some interaction; such annotations may be important features for supervised models learned for the task.

**Figure 1:**
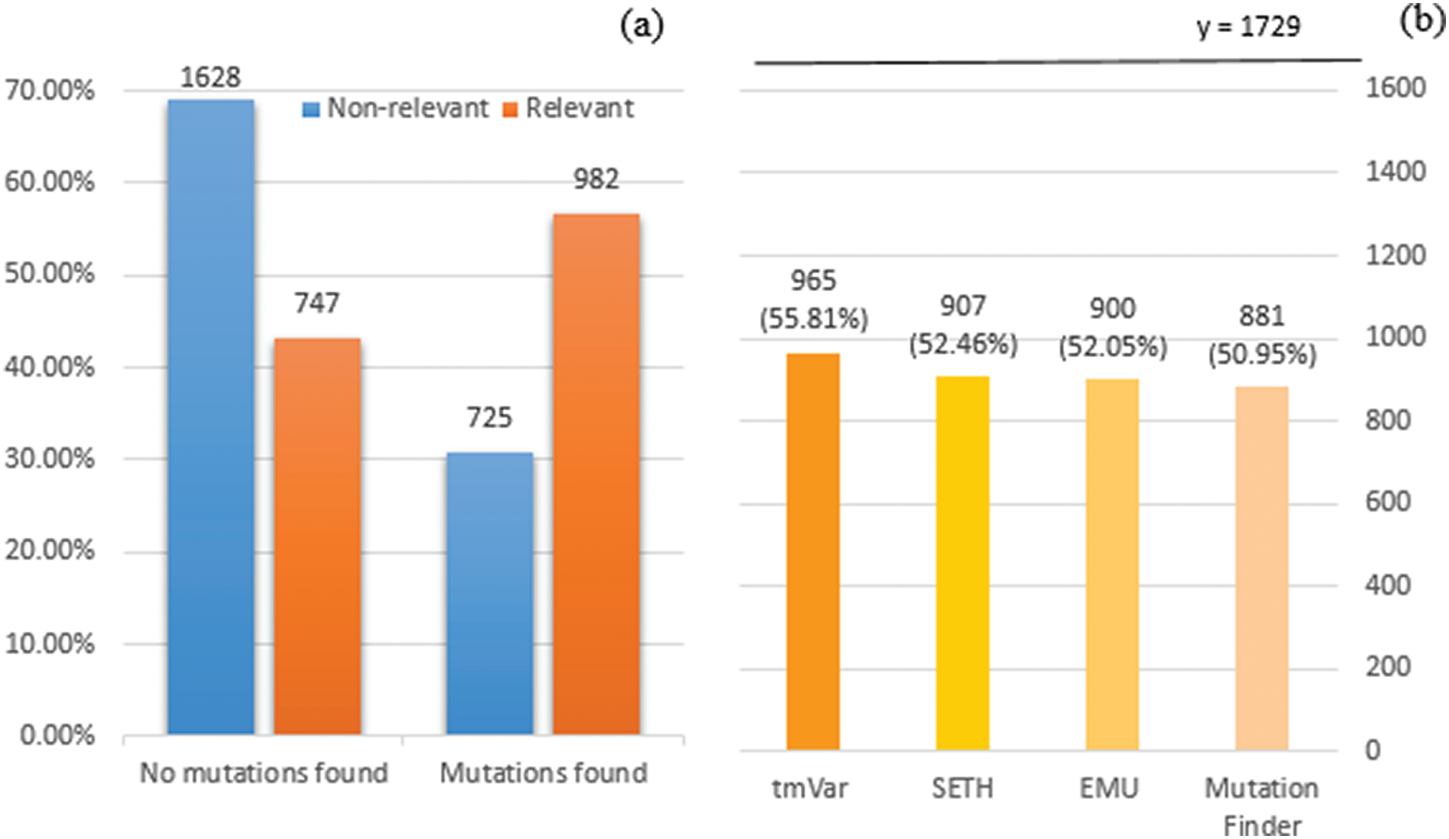
(a) Distribution of documents having a mutation identified by at least one of four mutation detection tools. Relevant: documents labelled as relevant for PPIm in the training set, otherwise non-relevant. Mutations found: documents have a mutation mention identified by at least one of the tools, otherwise no mutations found. The y-axis corresponds to the proportion relative to the relevant or non-relevant document collections, respectively. For instance, almost 70% of non-relevant documents (1628 out of 2353) have no detected mutation mentions. (b) Distribution of relevant documents having mutations identified by individual tools; y = 1729 is the total number of relevant documents, in which we would expect to have at least one mutation mentioned per task definition.

We employed tmVar (4), SETH (13), EMU (14) and MutationFinder (15) to recognize mutations in the training set. These tools have been used in a range of applications, such as evidence attribution for biological database curation (16) and genotype–phenotype extraction for precision medicine (17). The tools have been shown to have complementary coverage (18). Figure 1 presents the corresponding results: 1(a) shows the number of documents having mutations identified by at least one tool, whereas 1(b) specifically shows the number of relevant documents having mutations identified by individual tools. Overall it shows that relevant documents include identified mutation mentions more often than non-relevant documents, 56.8% vs 30.8% (and in fact, relevant documents could be expected to have 100% in reality), which shows that mutation related information from BioNLP tools can be potentially important to distinguish relevant from non-relevant documents. Nevertheless, the performance of the tools is relatively low; in this task the best tool identifies mutations in only 56% of relevant documents. The main reason for this gap is that many mutations or interactions are mentioned through general references rather than precise descriptions of individual mutations: as an example, `mutagenesis’ is the only term describing mutations in the paper (PMID:20485264 from the training set); this general mention is not detected by the tools. This suggests the necessity of alternative approaches to complement tools identifying specific mutations.

**Figure 2:**
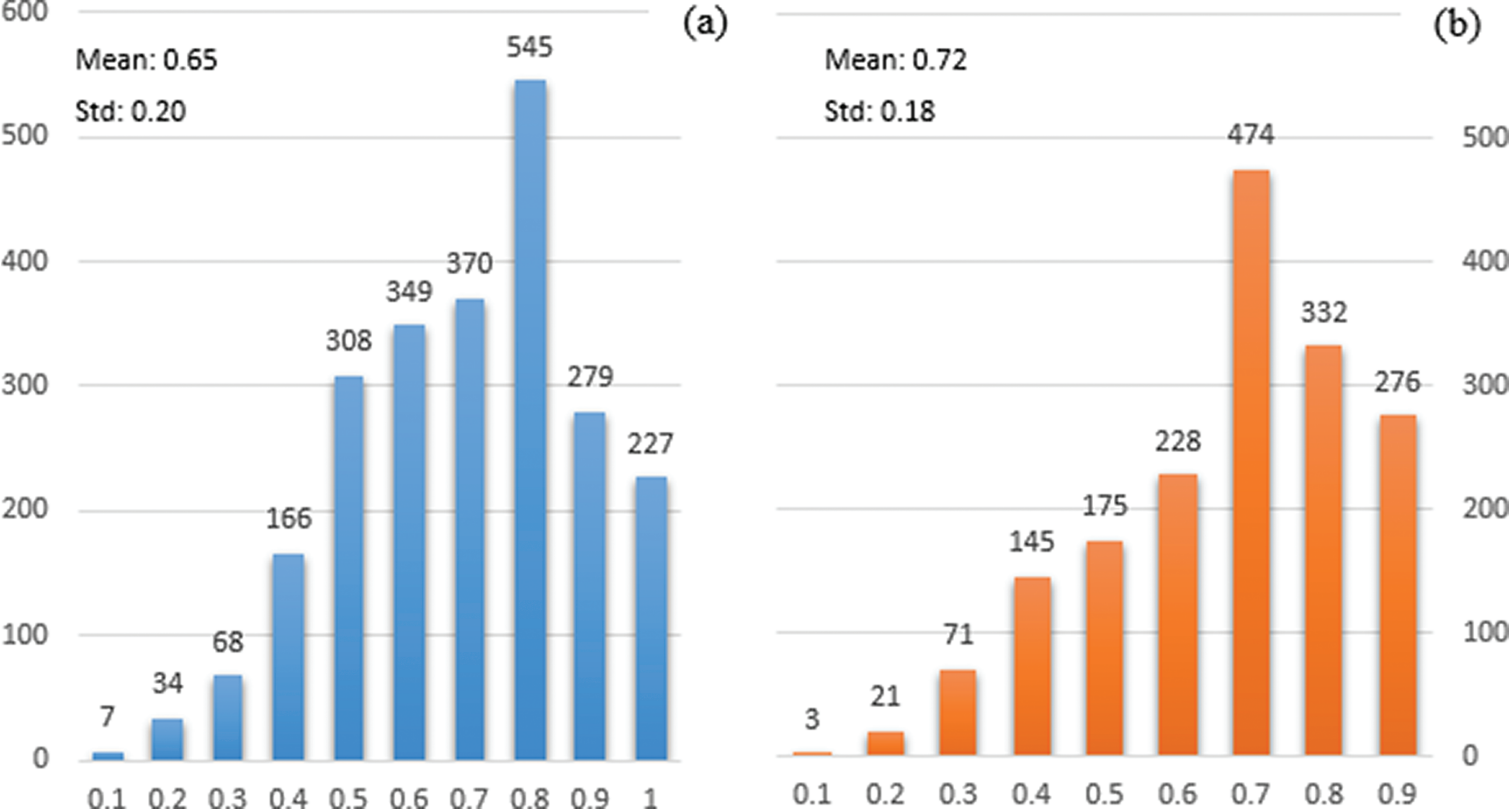
Distribution of the probability of documents having interactions identified by PIE the search. The x-axis displays the probability score output by PIE the search (normalized to [0, 1]); a higher score indicates higher probability of having interaction. The y-axis reflects the number of documents. (a) and (b) represent the distribution for non-relevant (blue) and relevant documents (orange), respectively.

The exploration of protein interaction characteristics in the training set yields more consistent results. We show in Figure 2 the result of applying PIE `the search’ as the representative BioNLP tool for interaction extraction. Figure 2(a) and (b) represent the probability score distribution for non-relevant and relevant documents, respectively. Similarly to the mutation characteristics, relevant documents on average have higher probability score; however, still over 37% of relevant documents have a relatively low probability score, less than 0.7. Given that PIE `the search’ has demonstrated very high precision at high confidence, and overall good F1-score of ∼0.62 for PPI extraction in prior studies, it is surprising not to see a larger proportion of the PPIm-relevant documents with high-confidence extractions of PPIs.

### Relation extraction

In this task, the goal is to extract interacting gene pairs from a document, where that interaction is affected by a mutation. Towards this goal, the task offers a collection of PubMed documents with relation annotations and a limited set of entity annotations. We illustrate these details with a sample sentence taken from the document with PMID:10037723, with a given PPIm relation annotated for the protein pair *(4292, 5395),* where these numbers correspond to the normalized gene identifiers for the genes `hMLH1’ and `hPMS2’.

`Interestingly, two HNPCC missense alterations (Q542L and L582V) contained within the consensus interaction region displayed no effect on interaction with hPMS2, suggesting that they may affect other functions of hMLH1.'

The entities mentioned in the above text, such as the disease HNPCC, the mutation Q542L and the gene hPMS2, are shown highlighted. Note that the annotations of PPIm relations provided in the data are document-level rather than annotated to specific mentions of genes in the text. This means that there is no guarantee that the two protein/gene mentions for a PPIm relation co-occur in the same sentence, and we do not know precisely where in the text the PPIm relation is expressed.

Our approach to relation extraction is to cast it as a supervised classification task over pairs of entity mentions (full details of our approach will be provided below). From the positively classified set of entity mention pairs, we map each entity mention to its equivalent normalized entity identifier and output pairs of entity identifiers that are in a relation. Therefore, for the PPIm relation extraction task, we must first perform entity recognition of gene mentions in the text, along with normalisation to NCBI Gene identifiers. We use the relation annotations provided in the document for training our classifier, but we found that the entity annotations provided in the task dataset are not comprehensive. For example, the protein mention `hMLH1’ in the above sentence is not annotated in the dataset. Further, the gene-annotations provided in the PPIm dataset are limited, as only those genes that participate in a PPIm relation are annotated. Therefore, we used GNormPlus in our earlier work (1), to get a broader set of gene annotations. In this work, we extend our investigation to include another entity annotator, namely the Pubtator web API (21, 22) and explain in detail how the different entity annotators differ from each other.

### Differences in entity annotators:

In our original work for the shared task in BioCreative-VI Track 4, we started with a clean slate approach by stripping all existing entity annotations from the training set. We then used GNormPlus (15) as sole entity annotator over the training and test datasets. In the current work, we investigate the impact of different annotation tools, starting with the default set of entity annotations provided in the task datasets (referred to as `Task annotations’ hereafter), GNormPlus and then Pubtator. The Task annotations are created manually and are of gold standard but do not represent a realistic entity annotation scheme. This is because they only contain annotations of those entities that participate directly in a PPIm relationship. In other words, the Task annotation scheme assumes knowledge of PPIm relations in the document and further encodes this knowledge by leaving out the annotations of entities that do not participate in any PPIm relation. In contrast, the external entity annotation tools, namely GNormPlus and Pubtator, represent more realistic annotation schemes, although they too are affected by errors. Our main motivation to include Pubtator in the current work is because it offers annotations for other event types such as mutations (incorporating the tmVar tool studied above), species names and chemicals, whose modeling can likely improve the relation extraction performance. We note that Pubtator too uses GNormPlus internally for gene annotations. However, there is a difference in the versions: Pubtator uses an older version of GNormPlus than is currently available for download. In this article, when we refer to GNormPlus, we mean the latest version of GNormPlus (15). An entity annotation can be considered as a triple (document id, character span, entity id). The output of applying the entity annotators is a set of entity annotations, which can be directly merged (union) when the underlying text span does not overlap. Therefore, integrating the non-gene annotations from Pubtator is straightforward, but it is not clear as to how to combine different entity annotations that overlap with each other. This is illustrated in this sample text from document PMID:17724026:

*`Immunoprecipitations were performed in myocytes expressing PKCzeta using PKC phospho-motif antibodies to determine the phosphorylation of cTnI, cTnT, tropomyosin, myosin-binding protein C, and desmin.’*

For the above sentence GNorm Plus annotates the substring `myosin-binding protein C, and desmin’ as a gene, but the corresponding Task annotation is just for the substring `desmin’. These differences can impact the relation extraction pipeline, such as entity masking steps, tokenisation, parsing and the overall feature representation of the sentence. Entity annotations can also differ on the entity ID, which is harder to resolve, as illustrated in this sample from the document PMID:10067897:

`Together, these protein-DNA and protein-protein interactions define the general principles by which homeotic proteins interact with Extradenticle (or Pbx1) to affect development along the anterior-posterior axis of animals.’

**Figure 3:**
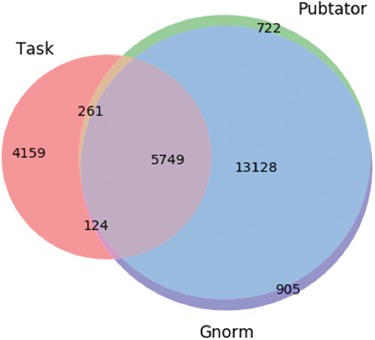
Venn diagram showing the differences in the gene entity annotations over the PPIm dataset. An entity annotation is regarded as a triple (document id, character span, entity id). The three sets are the set of annotations given in the task dataset (Task) and from the two entity annotators namely Pubtator and GNormPlus (GNorm).

Here, the character span `Pbx1’ is annotated with id 32567 by Pubtator and with id 5087 by GNorm. Gene 32567 relates to an extra denticle in Drosophilia (see https://www.ncbi.nlm.nih.gov/gene/32567) and 5087 (see https://www.ncbi.nlm.nih.gov/gene/5087) is a gene that influences skeletal programming in mammals, but these two different genes share a common name `Pbx1’; this leads to the confusion. The extent of differences in the different annotation tools are illustrated in Figure 3.

### Ensemble of entity annotators

We followed a simple heuristic approach to consolidate the entity annotations from the different sources. We include all non-gene entity annotations from Pubtator. When two entity annotations *e1*, *e2* overlap, i.e. if they refer to common text segments in a document, they are combined into a single annotation, using the following guidelines:

1. Take the minimal superstring that encompasses the character spans of both *e1* and *e2* as the character span of the resultant entity annotation.
2. We prioritize the annotations in the following order: Task, GNorm Plus and Pubtator. For overlapping annotations, we take the gene id from the annotation source with higher priority.

A summary of the distribution of entity annotations in the Relation Extraction Dataset is presented in Table 1.

**Table 1:** Distribution of entities as recognised by different entity annotators in the PPIm dataset

Count	Annotator	Training set	Test set
Number of documents	-	597	632
Number of PPI relations	Task	760	868
Gene annotations	Task	8832	1461
	GNorm	8677	11229
	Pubtator	8801	11059
	Annotator ensemble	10897	11665
Mutations	Pubtator	557	1722
Species	Pubtator	1102	948

The limitation imposed by entity annotators on the maximum relation extraction recall achievable can be seen in Table 2.

**Table 2:** Maximum recall achievable by our relation extraction system with different entity annotation schemes

Entity annotation	Maximum recall achievable by relation extraction
GNormPlus	55.95
GNormPlus + Pubtator	56.30
Task annotations	1.0
Annotator ensemble	1.0

### Summary of the training set study

The examination of the training set characteristics gives three implications: (i) entity recognition is important for both tasks; for example, entities recognized by BioNLP tools can be important features to differentiate relevant and non-relevant documents; (ii) while entity recognition by BioNLP tools is important, complementary approaches are necessary given that many mutations and interactions of the relevant documents cannot be identified; and (iii) the proposed model should have a high recall for both tasks. Arguably, recall is not an effective evaluation metric in general information retrieval domains (23) and is not in bioinformatics domains either, such as biological sequence database retrieval (24). In this task, however, it is critical since the standard BioNLP tools have a relatively low recall in entity recognition as shown above. Arguably, recall is even more important for the document triage task; the relation extraction system is reliant on having previously identified relevant documents.

## Methods

In this section, we describe the models we developed for the two tasks that form part of BioCreative VI Track 4.

### Document triage models

For document triage, we develop a range of features and quantify the importance of the features via a feature contribution study using a simple logistic regression classifier. The above analysis on the training set shows that while mutations and interactions are important features to distinguish relevant and non-relevant documents, using BioNLP tools alone cannot find many relevant mutation and interaction mentions. We therefore use other complementary approaches to develop features, as described below.

### Feature engineering

We develop a set of features based on a variety of characteristics of the text, according to four primary aspects. We calculate distinct features for both sentences and paragraphs (**structure-based)**, considering both terms identified as key biological concepts and any word (**perspective-based**) and then considering either individual occurrence, a co-occurrence of two or three terms (**occurrence-based)**. Each of these is represented in terms of various quantities (**count-based**).

The **structure-based** aspect defines features based on the structure of the document; in this task, there are two relevant structures: features developed from **paragraphs** (that is, the title and the abstract) and **sentences** respectively (that is, each sentence in the paragraph). For instance, the number of genes in total identified in a paragraph by BioNLP tools is a feature derived from paragraph-level analysis, whereas the number of sentences having mutations identified by BioNLP tools is a feature derived from sentences.

The **perspective-based** aspect defines features according to the perspective through which key terms in the texts are identified: based on **BioNLP tools**, matched to a pre-defined **term list**, or **both**.

In BioNLP systems, term lists that capture important entities are often used to complement automatic BioNLP tools (25–27). We chose three BioNLP tools: tmVar (4) to detect mutations (although Figure 1 shows the ensemble of four tools has the highest number of mutations detected, tmVar alone detects nearly all of them and others are almost strict subsets of mutations identified by tmVar), GNormPlus (12) to detect genes and interactions (if the number of genes detected is greater than or equal to two (2)) and PIE the search (13) to output the probability score. Given that PIE the search outputs the probability score rather than specific entities, we use the score as a separate feature.

Given that BioNLP tools cannot find most of the entities, as we showed above, term lists are particularly useful in this task. We develop three term lists for `mutations’, `interactions’ and `degrees’ (changes to interactions due to mutations), respectively.

The `mutation’ term list contains terms which were based on the observations from existing mutation resources such as the Variome corpus (28). The list is split into `strong’ and `weak’ terms. `Strong’ terms are mostly unambiguously used to describe mutations in the literature: mutant-based terms (`mutation, mutant, and mutants/variant*’), `*delete’*, `*insertion*’, `*substitution*’, −/−, +/−,* the terms *`*polymorphism’, `SNP’, `lesion’*,* mutagenesis-based terms (`mutagenesis, mutagenic, mutagenetic and mutagenesis/delete*’*)*, `*deleterious’ and `variant’. The `weak’ terms considered include `change’, ‘exchange’, ‘damage’, ‘remove’, ‘replace’, ‘disorder’, ‘deficiency’, ‘virulence’ and ‘truncation’. While they are used to describe mutations, they may also be used in other contexts; for instance, the term change can be used in `DNA base change’ but can also be used in ‘changes in the distribution’. We process strong and weak terms separately when developing features: ‘strong’ terms themselves can indicate the mentions of mutations directly; in contrast, ‘weak’ terms can indicate the mentions of mutations only if the amino acids (such as Alanine and Arginine) are co-mentioned in the text. We use the amino acid patterns provided in a previous study (29) to detect the amino acids.

The `interaction’ term list contains 30 terms describing interactions frequently in literature, such as ‘interact’, ‘complex’, ‘bound’, ‘bind’, ‘regulate’, ‘kinase’, ‘acetylation’, ‘phosphorylation’ and many others.

We further create a list of 23 `degree’ terms that are indicative of the impact a mutation may have on a protein interaction. For example, `increase’ is a term often used to describe that the presence of a mutation increases the level of interaction, including ‘degrade’, ‘decrease’, ‘strengthen’, ‘enhance’, ‘reduce’ and ‘impair’. These degree terms are derived from molecular interactions ontologies (from www.ebi.ac.uk/ols/ontologies/mi). All the term lists are available in the repository. For the terms mentioned above, we applied stemming to the original text and then did exact matching to identify them in the texts.

The **occurrence-based** aspect is based on whether interactions, mutations and degrees appear in three ways: `individual’, where interactions or mutations exist alone; `co-occurrence’, where interactions and mutations appear together within the relevant structural scope and `triplet’, whether a mutation–degree–interaction triplet appears. Since documents relevant for the PPIm task must have both interactions and mutations mentioned, features derived from this category can potentially distinguish positive documents from negatives.

Quantitative features are determined for each combination of the above aspect using a choice from the **counting-based** aspect. If an entity or a co-occurrence of two entities is detected from a single perspective, i.e. either based on BioNLP tools or term lists, we count the `total’ number of mentions or the number of `unique’ entities. In contrast, if an entity is detected through both perspectives, there are three possibilities: (i) `intersection’: for example, the number of entity or co-occurrence mentions identified by both BioNLP tools and the term lists; (ii) `union*’*: the count of entities or co-occurrences where either approach has identified them; and (iii) `complement’: for example, GNormPlus finds two genes and the mutation term lists finds a mutation in a sentence; in this case, BioNLP tools and term lists complement each other.

We incorporate two additional quantitative features: (i) the probability score from PIE the search is a separate feature as mentioned above and (ii) the `impact’ of a sentence based on the co-occurrence of interactions and mutations using the simple algorithm shown in Algorithm 1. In this case, the impact score is a real-valued number between 0 and 1.

**Algorithm 1:**
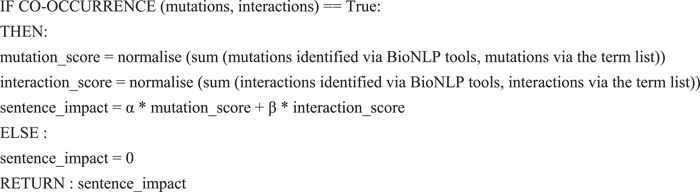
The algorithm measures the 'impact' of a sentence. If there is a co-occurrence relationship, identified either using intersection, union or complement as mentioned in Counting-based category, it firstly calculates the mutation and interaction scores by summing the total mentions and normalising to [0, 1]. Then it calculates the impact score based on the weight of mutation and interaction scores (α and β). The default weights are 0.5, meaning that mutations and interactions are equally important.

Overall, we develop features by combining choices within these different aspects. For example, the number of sentences containing both interactions and mutations identified by BioNLP tools follows the `Sentence-BioConcept-Co-occurrence-Total’ aspect settings.

**Table 3:** Feature contribution study results. Each set of features in a row is added to the existing feature set; for example, term paragraph individual features represent the new features added to the baseline features. The description of a set is consistent with the description of feature aspects. 2-gram: every two sentences. Fold 1 represents the first fold using 10-fold cross-validation, same for other folds. Table 4 provides the detailed descriptions of the best feature set found in the feature contribution study

	Fold 1	2	3	4	5	6	7	8	9	10	F1 mean (±std)
Baseline	0.6390	0.6418	0.6457	0.6118	0.6435	0.6577	0.6611	0.6461	0.6286	0.5714	0.6347 (±0.0262)
Term paragraph individual features (S1)	0.6796	0.6523	0.7120	0.6538	0.6832	0.7143	0.6610	0.7119	0.6991	0.6386	0.6806 (±0.0281)
Term paragraph co-occurrence features (S2)	0.6839	0.6541	0.7065	0.6474	0.6814	0.7128	0.6629	0.7139	0.7012	0.6488	0.6813 (±0.0266)
BioNLP paragraph individual features (S3)	0.6753	0.6542	0.7215	0.6538	0.6868	0.7363	0.6685	0.7155	0.7012	0.6667	0.6880 (±0.0292)
BioNLP paragraph co-occurrence features (S4)	0.6753	0.6584	0.7215	0.6538	0.6923	0.7363	0.6722	0.7135	0.6972	0.6667	0.6887 (±0.0281)
Term sentence individual feature (S5)	0.6753	0.6522	0.7139	0.6431	0.6904	0.7415	0.6685	0.7155	0.7012	0.6588	0.6860 (±0.0318)
Term sentence co-occurrence feature (S6)	0.7386	0.6847	0.7538	0.6688	0.6885	0.7558	0.7193	0.7348	0.7169	0.6586	0.7120 (±0.0349)
BioNLP sentence individual feature (S7)	0.7386	0.6828	0.7570	0.6731	0.6885	0.7609	0.7158	0.7348	0.7169	0.6606	0.7129 (±0.0353)
BioNLP sentence co-occurrence feature (S8)	0.7320	0.6826	0.7468	0.6688	0.6921	0.7520	0.7123	0.7313	0.7169	0.6727	0.7108 (±0.0303)
Term & BioNLP sentence feature (S9)	0.7284	0.6903	0.7558	0.6730	0.6957	0.7572	0.7033	0.7308	0.7234	0.6607	0.7118 (±0.0328)
Term & BioNLP sentence triplet feature (S10)	0.7273	0.6825	0.7538	0.6730	0.6940	0.7461	0.7139	0.7534	0.7077	0.6786	0.7130 (±0.0311)
Term & BioNLP sentence triplet 2-gram feature (S11)	0.7273	0.6807	0.7661	0.6752	0.6959	0.7532	0.7174	0.7520	0.7099	0.6806	0.7158 (±0.0332)
Full feature set plus boosting	0.7571	0.7086	0.7646	0.7108	0.7059	0.7809	0.7500	0.7627	0.7251	0.6899	0.7355 (±0.0311)

### Feature importance

Since there are many features created in this approach, we perform a feature contribution study to find the best combination. To create a baseline model, we first performed simple text processing on the original document, including case-folding, tokenising, removing stop words and punctuation and stemming, using the NLTK package (30). We then create tf-idf matrices for processed tokens in each document. Finally, a logistic regression model is built using the tf-idf matrices as features with Scikit-learn (31). Tf-idf weighting, quantifying the importance of a term based on its frequency as well as penalising very frequently occurring terms across the documents, is widely used in text classification and information retrieval (32, 33); similarly, logistic regression is often used as a baseline model for text classification since it is robust to sparse matrices (tf-idf matrices are often sparse such that a small proportion of terms have very high frequency whereas other terms have negligible occurrences) and outputs the probability score of the classification (34, 35). This baseline achieves a 63.5% F1 score, which is consistent with the baseline results reported by the task organizers (8).

After creating the baseline model, we introduce additional features based on the feature aspects and measure the updated performance. The results in Table 3 show the importance of different feature sets and the best feature set using boosting logistic regression gives an ∼10% increase on the F1 score as well as only increases the standard deviation by ∼0.5%; the detailed description of these best features are shown in Table 4. We find the following:

1. Term lists are effective, giving an increase of ∼4% F1 score even when considering only paragraph level;
2. Co-occurrence features are more effective at sentence level; for example, both co-occurrence features from BioNLP tools and term lists at paragraph level only give ∼0.8% additional improvement, whereas the counterparts at sentence level increase the score by ∼3%;
3. Using term lists and BioNLP tools together can further improve the F1 score by 0.5%. Co-occurrence (mutations and interactions) often occur within one sentence, while triplets (mutation–degree–interaction) often occur in two sentences.

**Table 4:** Detailed descriptions of the best feature sets found via the feature contribution study in Table 3

Feature set	Feature ID	Description	Aspects
S1	F1	Number of interactions identified in total across the paragraph by term lists	Paragraph-Term-Individual-Total
F2	Number of unique interactions identified across the paragraph by term lists	Paragraph-Term-Individual-Unique
F3	Number of mutations identified in total across the paragraph by term lists	Paragraph-Term-Individual-Total
F4	Number of unique mutations identified across the paragraph by term lists	Paragraph-Term-Individual-Unique
S2	F5	Number of interactions and mutations in total across the paragraph by term lists if co-occurrence exists	Paragraph-Term-Occurrence-Total
F6	Number of unique interactions and mutations across the paragraph by term lists if co-occurrence exists	Paragraph-Term-Occurrence-Unique
S3	F7	Number of genes identified in total across the paragraph by BioNLP tools	Paragraph-BioConcept-Individual-Total
F8	Number of unique genes identified across the paragraph by BioNLP tools	Paragraph-BioConcept-Individual-Unique
F9	Number of mutations identified in total across the paragraph by BioNLP tools	Paragraph-BioConcept-Individual-Total
F10	Number of unique mutations identified across the paragraph by BioNLP tools	Paragraph-BioConcept-Individual-Unique
F11	The probability of the paragraph containing interactions by BioNLP tools (using PIE the search)	Paragraph-BioConcept-Individual-Total (probability)
S4	F12	Number of interactions and mutations in total across the paragraph by BioNLP tools	Paragraph-BioConcept-Occurrence-Total
S5	F13	Number of sentences containing mutations by term lists	Sentence-Term-Individual-Total
F14	Number of sentences containing interactions by term lists	Sentence-Term-Individual-Total
S6	F15	Number of sentences containing both interactions and mutations by term lists	Sentence-Term-Occurrence-Total
S7	F16	Number of sentences containing mutations by BioNLP tools	Sentence-BioConcept-Individual-Total
F17	Number of sentences containing genes by BioNLP tools	Sentence-BioConcept-Individual-Total
S8	F18	Number of sentences containing both interactions and mutations by BioNLP tools	Sentence-BioConcept-Occurrence-Total
S9	F19	Number of sentences containing both interactions and mutations either by term lists or BioNLP tools	Sentence-Both-Occurrence-Union
F20	Number of sentences containing both interactions and mutations either by term lists or BioNLP tools using complementary approach	Sentence-Both-Occurrence-Complement
S10	F21	Number of sentences containing mutation-impact-interaction triplets by term lists	Sentence-Term-Triplet-Total
F22	Number of sentences containing mutation-impact-interaction triplets by term lists or BioNLP tools	Sentence-Both-Triplet-Union
F23	Number of sentences containing mutation-impact-interaction triplets by term lists or BioNLP tools or one another	Sentence-Both-Triplet-Complement
S11	F24	Same as F17, but on number of sentence 2-grams	Sentence-Term-Triplet-Total
F25	Same as F18, but on number of sentence 2-grams	Sentence-Both-Triplet-Union
F26	Same as F19, but on number of sentence 2-grams	Sentence-Both-Triplet-Complement
F27	Same as F22, but using average probability of sentences instead of number of sentences	Sentence-Both-Triplet-Complement (probability)

We use the best feature set determined through this study, together with tf-idf term weightings, to train boosting logistic regression, Support Vector Machine (SVM) and Random Forest models for classification.

### Relation extraction models

For PPIm relation extraction in the shared task, we explored two methods: (i) graph kernels based on dependency parsing of sentences; and (ii) a co-occurrence based relation extraction system. In this work, we have extended our evaluation to include a feature-based classifier using word bigrams. These methods are described in detail in this section.

### Graph kernel-based approach:

We used a two-stage approach of dataset preprocessing to generate candidate gene pairs, followed by a supervised classifier that detects PPIm relations amongst the candidate entity pairs. These steps are described below.

‘Preprocessing’: In the pre-processing stage, we use the Turku Event Extraction System (TEES) (36) to parse each document into a set of sentences and the dependency graph representation of these sentences. For example, considering the previous example again:

… LAF1, an R2R3-MYB factor, interacts with HFR1, a basic helix-loop-helix (bHLH) factor, and this interaction is abolished by the R97A mutation in the LAF1 R2R3 domain. ... This result indicates that LAF1 and HFR1 function in largely independent pathways. LAF1, an R2R3-MYB factor, interacts with HFR1 …

**Figure 4:**
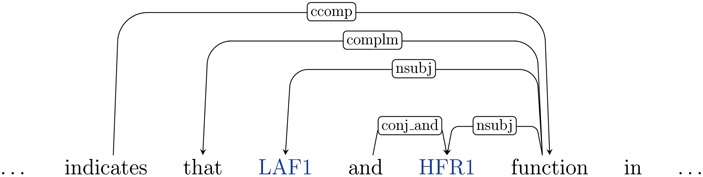
Illustration of the (partial) dependency graph for the sentence `This result indicates that LAF1 and HFR1 function in largely independent pathways’. The entities (genes) are shown in blue.

The dependency graph representation of a snippet from this above text is illustrated in Figure 4.

Next, we use the entity annotations corresponding to this document, to obtain a list of gene mentions in this document, such as LAF1 and HFR1. All possible pairs of genes are generated as candidate relations. Note that the two mentions in a pair may be separated by zero or more sentence boundaries. We refer to these as sentence-level relations and non-sentence relations, respectively. Sentence-level relations are processed using the dependency graph of the underlying sentence. For example, the entity pair (LAF1, HFR1) is represented by the dependency graph shown in Figure 4. For non-sentence relations, we pick the two dependency graphs of the sentences containing the relevant entity mentions. These two graphs are connected by inserting a special edge between their root nodes and the resultant graph is used to represent the cross-sentence entity pair, following the approach of our prior work (50).

*‘*Entity masking’: Given an entity mention pair and its dependency graph representation, we replace the labels of the nodes corresponding to the two Proteins with generic strings such as ‘Protein1’ and ‘Protein2’ in the dependency graph. These are the nodes whose corresponding tokens in the sentence overlaps with the character span of given entity mentions. Entity mentions in the sentence that are not the primary arguments are replaced with generic strings such as ‘Protein_Other’, ‘Mutation_Other’, ‘Species_Other’ and ‘Chemical_Other’, based on the entity type to which it belongs to. This step is shown to be effective in improving generalisation in prior event extraction studies (37). Also, these special strings serve the purpose of imparting the entity information to the dependency graph, by discriminating the main event arguments (gene pairs) from the rest of the tokens. These modified dependency graphs are used as examples for a graph kernel-based classifier that is described below. Including additional entity types such as mutations, was found to help improve the performance of the relation extractor. In the next step, we prune the candidate list of entity pairs as described below.

**Table 5:** The candidate examples generated for relation classification with different entity annotation schemes

Type	Entity annotator	Training set		Testing set	
		**Positive examples**	**Negative examples**	**Positive examples**	**Negative examples**
Sentence-level relations	Task	4115	532	264	152
	GNormPlus	1709	4697	2829	5780
	GNormPlus + Pubtator	1783	4822	2865	5835
	GNormPlus + Pubtator + Task	4058	4533	3097	6030
Non-sentence level relations	Task	412	162	556	178
	GNormPlus	89	4616	321	10105
	GNormPlus + Pubtator	89	4734	338	10190
	GNormPlus + Pubtator + Task	430	5317	872	11719

*‘*Filtering self-relations’: We used a heuristic rule of filtering out all self-relations, i.e. we disallow a PPIm relation between identical gene IDs. We were motivated by the intuition that a gene or protein typically does not interact with itself. We found that self-relation filtering improves the F-score substantially on both the training set (0.2554 to 0.2834) and test set (0.2960 to 0.3355). However, it also limits the recall performance of our relation extractor, as 6% of relations in the test set are self-relations. For example, in the document id 17074813, titled ‘Differential regulation of B-raf isoforms by phosphorylation and auto-inhibitory mechanisms’, the gene B-raf with NCBI ID 673, contains a valid PPIm self-relation annotation (673, 673).

In the final step, the resulting set of candidate entity pairs are input to a binary classifier for training the model and at test phase for identifying relations. The statistics of the examples generated for classification are listed in Table 5.

*‘*Relation classification*’:* Given entity mention pairs and the corresponding dependency graphs from the preprocessing step described above, we train a supervised classifier as follows. For every entity mention pair in the training set, we look up on their corresponding entity ids (NCBI gene id) in the relation annotations to determine if there is a PPIm relation. Such examples are labelled positive and the rest are labelled negative. We separated the sentence level relations and non-sentence level relations into two separate classification pipelines and trained two separate binary SVM classifiers for these pipelines. We used the Approximate Subgraph Matching (ASM) kernel with the SVM classifiers, as ASM kernel is designed primarily to work with edge labelled dependency graphs for relation classification. The ASM kernel translates an input graph into a high-dimensional feature representation. More details about the ASM kernel are available in (53). We implemented our classifier with the scikit-learn (31) library in Python, using the SGD classifier with hinge loss. The class weights were set to 2:1 for the sentence classifier and 6:1 for the non-sentence classifier. In the final stage, the entity mention pairs generated from the PPIm test set are classified and from the positively labelled entity mention pairs, we extract their corresponding entity ids (NCBI gene ids) and output the union of these as the document level relations.

*`*Document relevance score and mutation context*’:* The document triage task associates each document with a score that represents the probability of it holding PPIm relations. We experimented with using this relevance score as an additional feature for the relation extraction task. We also experimented with using a set of specialized terms to help recognize mutation mentions in a sentence with the hypothesis that it improves relation extraction performance. This is the same list of terms that was found to improve document triage classification performance and is described in detail in the feature engineering section of document triage classification above.

### Co-occurrence-based PPIm extraction

In the co-occurrence based approach, we use GNormPlus (12) for protein entity recognition and LingPipe (http://alias-i.com/lingpipe/index.html) for delimiting sentences. We begin by identifying all pairs of protein mentions that occur within a single sentence.

**Table 6:** Document triage task performance over the training set using 10-fold cross-validation

Model	Training time (sec)	Prediction time (sec)	Ranked precision	Precision	Recall	F1
Baseline	0.5885 (±0.0949)	0.0006 (±0.0001)	0.6839 (±0.0303)	0.6485 (±0.0353)	0.6229 (±0.0353)	0.6347 (±0.0262)
LR (boosting)	26.7562 (±4.0662)	0.0500 (±0.0133)	**0.7580** (±0.0262)	0.7058 (±0.0313)	0.7684 (±0.0368)	**0.7355** (±0.0311)
SVM	65.8464 (±0.8162)	1.1460 (±0.0398)	0.7479 (±0.0287)	**0.7097** (±0.0305)	0.7102 (±0.0443)	0.7095 (±0.0322)
RF	829.0522 (±54.4509)	8.4559 (±0.1722)	0.7457 (±0.0370)	0.6651 (±0.0322)	**0.7946** (±0.0243)	0.7236 (±0.0227)

Our approach then applies three heuristics to filter protein pairs:

1. Filter out any self-relationships (i.e. a protein cannot interact with itself), as for the graph-based approach.
2. Given that an abstract describes at least one PPIm relationship, the more mentions a protein pair has in such an abstract, the more likely it is that the pair participates in a PPIm relationship. We considered the number of sentences N containing a given protein pair to define a threshold to extract protein pairs.
3. A default rule that applies if a protein pair falls below the threshold but is the only pair mentioned in a sentence that also contains the word ‘interact’. The choice of ‘interact’ as the trigger word was based on it being the most frequent term used to express protein interaction relationships in the training set.

We set the co-mention sentence threshold N for heuristic H2 empirically, based on the best F-score on the training set for N ranging from 1–4, selecting *N* > = 3, requiring a co-occurring protein pair to appear in three or more sentences in the abstract.

## Results

We present the results from cross-validation testing over the training set, as well as the results on the latest official test set for both tasks.

### Document triage

The experimental results for document triage are collectively shown in Tables 6 and 7. We perform 10-fold cross-validation to evaluate the performance of the models. We report four evaluation metrics: mean average precision (the precision based on the rankings of returned relevant documents), precision (the proportion of classified relevant documents that are indeed relevant), recall (the proportion of correctly classified relevant documents over the total number of relevant documents) and F1 (the harmonic mean of precision and recall). The choices of these evaluation metrics are based on the use cases of the document triage task. The task needs to support retrieval of relevant documents for biocurators or biologists: the model ideally should return the highly relevant documents that it classifies at the top so that biocurators can examine the top documents more carefully without exhaustively looking for all the returned documents. Ranked precision is an important information retrieval metric that quantifies this criterion (38). Likewise, the model should also find most of the relevant documents over the entire search space in a precise manner, where precision and recall are used as the primary measures in classification tasks.

**Table 7:** Document triage task performance on the test set

Model	Ranked precision	Precision	Recall	F1
Baseline	0.6329	0.5852	0.6733	0.6262
LR (BOOSTING)	**0.6822**	0.5783	0.7713	0.6610
SVM	0.6721	**0.5936**	0.7116	0.6473
RF	0.6744	0.5361	**0.8849**	**0.6677**

**Table 8:** Relation Extraction Performance with different modes of entity annotation. Performance measurements for the training set are based on 10-fold cross validation. Best results for training and test sets are highlighted, for entity recognition with standard BioNLP tools

Entity annotation	Relation extraction method	Training set			Test set		
		Precision	Recall	F1	Precision	Recall	F1
Oracle	ASM with sentence level relations only	0.8472	0.8112	0.8288	0.7605	0.2923	0.4223
+ Non-sentence relation extraction		0.8373	0.8830	0.8595	0.7632	0.8826	0.8186
+ Relevance info		0.8308	0.8883	0.8586	0.7665	0.8803	0.8195
+ Mutation terms		0.8346	0.8790	0.8562	0.7614	0.8849	0.8185
Co-occurrence method (N > = 3)		0.8755	0.6263	0.7302	1.000	1.000	1.000
							
GNormPlus	ASM with sentence level relations only	0.2997	0.2766	0.2877	0.3208	**0.3636**	0.3409
+ Non-sentence relation extraction		0.2987	0.2793	0.2887	0.3189	0.3636	0.3398
+ Relevance info		**0.3013**	0.2753	0.2877	0.3277	0.3544	0.3405
+ Mutation terms		0.2965	0.2819	**0.2890**	0.3277	0.3579	0.3421
Co-occurrence method (N > = 3)		0.1583	**0.8883**	0.2688	**0.4000**	0.3098	0.3492
							
GNormPlus+Pubtator	ASM with sentence level relations only	0.2832	0.2832	0.2832	0.3464	0.3556	**0.3509**
+ Non-sentence relation extraction		0.2821	0.2832	0.2827	0.3384	0.3567	0.3473
+ Relevance info		0.2808	**0.2846**	0.2827	0.3467	0.3383	0.3425
+ Mutation terms		**0.2849**	0.2819	**0.2834**	0.3312	0.3544	0.3424
Co-occurrence method (N > = 3)		-	-	-	-	-	-
							
GNormPlus+Pubtator +Oracle	ASM with sentence level relations only	0.4066	0.7699	0.5322	0.3147	0.6030	0.4136
+ Non-sentence relation extraction		0.4066	0.7726	0.5328	0.3118	0.6053	0.4116
+ Relevance info		0.4068	0.7686	0.5320	0.3107	0.6064	0.4109
+ Mutation terms		0.4052	0.7699	0.5309	0.3147	0.6018	0.4133
Co-occurrence method (N > = 3)		0.3061	0.2487	0.2744	0.6623	0.9954	0.7954
Co-occurrence method (N > = 9)		0.3961	0.1090	0.1710	0.9183	0.9954	0.9553

Table 6 shows the document triage performance together with the training and prediction time. We can see all the three models achieve higher performance than the baseline model, especially the boosting logistic regression and the random forest model having 9–10% higher F1 score. Also, the variance over the 10 interactions is only ~0.5% more than the baseline, showing that the models do not tend to overfitting. The three classification algorithms, interestingly, each achieve the highest result in some individual measure. The boosting logistic regression gives the best ranked precision, whereas SVM has the highest precision and the random forest model achieves the highest recall. These results show that the models can complement each other and can be used in specific tasks where one metric is more important than others. The boosting logistic regression achieves the overall best performance: in addition to the highest ranked precision, it also has the highest F1 score and the training and prediction time is the shortest. The random forest model is also a good choice because it achieves ~80% recall. Recall is arguably more important than precision in this specific task given that standard BioNLP tools can only achieve ~50% recall in the entity recognition step, so that biocurators will not miss any important documents to annotate.

Table 7 presents the document triage results from the test set. The Random Forest achieves over 88% recall. However, we notice that the performance overall decreases for all the models. While it is often expected that the performance of the models decreases in the testing set, we notice the changes in the results are not regular. Taking the random forest model as an example, its precision dramatically drops by 12% but the recall surprisingly increases by ~9%. Similarly, the boosting logistic regression sees a decrease of about 12% precision while the recall remains almost the same. These results indicate that the model has many false positives (FPs): classifying **non-relevant** documents as **relevant**. We performed a detailed error analysis to gain insight into this, presented below.

### Relation extraction

For relation extraction, we used the official evaluation script provided by the Track 4 organizers to measure the micro-averaged Precision, Recall and F1-score of our relation extraction system on the task test set. We also report the results using 10-fold cross validation on the training set. These are shown in Table 8. We measure the performance considering the different possible choices for entity annotation, namely only the annotations provided in the task data (Oracle), GNormPlus alone, GNormPlus and Pubtator together, and finally a combination of all the entity annotations available.

Among the different entity annotation schemes, task (oracle) annotations do not represent a realistic scenario, as only those entities that participate in a PPIm relation are annotated (ignoring, for instance, other proteins that participate in a PPI but not a PPIm). Therefore, the candidate entity pairs formed from these annotations already reflect a high bias towards a PPIm relation. This results in a high precision of 0.8472 and 0.7615 for the training and testing set, respectively, for the machine learning methods. The nature of task annotations is also such that, amongst the candidate entity pairs formed in the test set, most relations (556 out of 868) are found beyond a sentence boundary, resulting in poor performance for sentence level relation extraction. With non-sentence level relation extraction included, our machine learning methods improve to get an F-score of 0.8186 on the test set. In contrast, our simple co-occurrence-based method achieves a best F-score of 1.0 on the test set and 0.7302 on the training set. These scores reflect the skewed nature of oracle entity annotations.

The entity annotation schemes, namely Task annotations, GNormPlus and Pubtator, represent a more realistic entity annotation scenario, as these are standard automated BioNLP tools. Note that GNormPlus provides Gene Annotations alone, but Pubtator brings in other information such as annotations for Mutations and Species, which can benefit our machine learning models for relation extraction. We note that the best performance is achieved with the ensemble of Pubtator and GNormPlus for the machine learning methods, leading to an F-score of .3509. The co-occurrence approach was tested using Gene annotations from GNormPlus alone and attained an F-score of 0.2698 on the training set and 0.3492 on the test set.

We recall that GNormPlus and Pubtator still miss out a substantial number of entities in the PPIm dataset that are provided in the Task annotations. Based on these entity annotators alone, a relation extraction system can attain a maximum recall performance of 0.56 as shown in Table 2. A perfect entity annotator can be expected to recognize nearly all entities in the dataset and should allow for the recall performance to reach 1.0. Such an entity annotation scheme would help estimate the performance improvement attainable by an improved entity annotation scheme. Towards this goal, we take the ensemble of all annotators including the task annotations to produce an oracle entity annotator that would give the relation extraction system an upper bound of 1.0 recall. Under the oracle conditions, the F-score of machine learning methods improves from 0.2834 to 0.5328 in the training set and 0.3509 to 0.4136 in the test set. For the co-occurrence approach, the inclusion of task entity annotations results in the maximum possible recall of 0.9954 and an F-score of 0.9553. These tests confirm that the main bottleneck in our relation extraction system is in the entity recognition phase.

**Figure 5:**
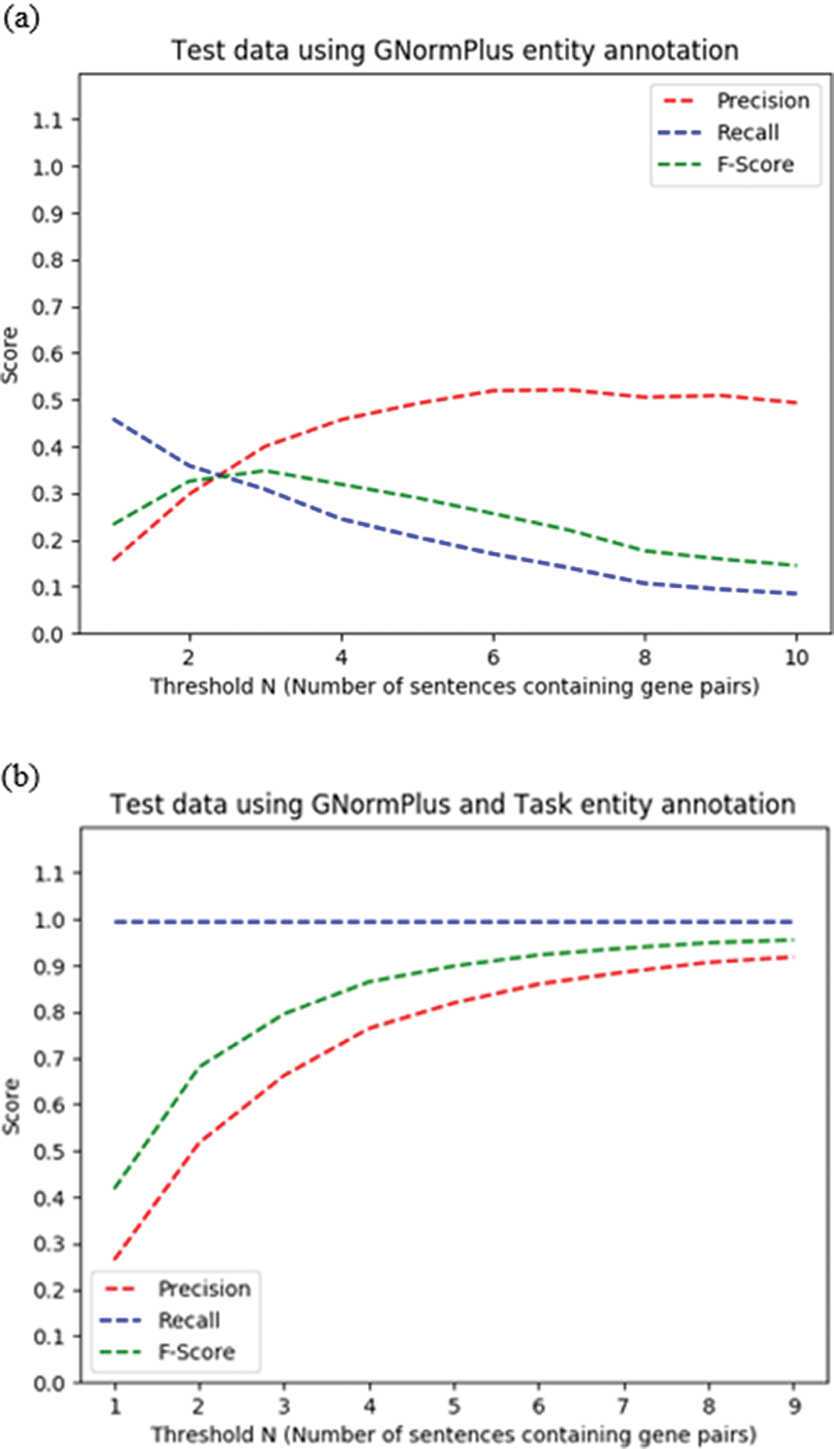
Impact of choice of threshold N = number of sentences containing a given protein pair on performance of heuristic co-occurrence approach on the test data set, for (a) automated protein/gene named entity recognition and (b) oracle protein named entity recognition scenarios.

We explore the threshold value *N* for the co-occurrence method in the test set in Figure 5. With the automatic GNormPlus-annotated protein entities, the best F-score occurs at N ≥ 3 and hence reflects the pattern that was observed in the training set. However, if we include the oracle gold entity annotations for the data the F-score is close to 1 at N ≥ 7, as shown. At such a high threshold, few protein pairs satisfy the co-mention sentence threshold, and the default rule applies in most cases.

To summarize, our relation extraction system attains a best F-score of 0.3509 using standard BioNLP tools for entity recognition, using a machine learning based approach. For the cross-validation performance over the training set, the machine learning approach achieves an F-score of 0.2890. Interestingly, our simple co-occurrence-based approach that does not use any linguistic features or machine learned model achieves a comparable F-score of 0.3492 on the test set. The standard BioNLP tools have low recall performance in the entity recognition phase, limiting the performance of our relation extraction system.

## Discussion

In this section, we present an in-depth error analysis and discussions for both tasks. Overall, we find mutation characteristics have different impacts on both tasks: for document triage, the model performance is dramatically decreased due to the significantly different mutation characteristics between the training and testing set; in contrast, for relation extraction, recognising mutations facilitates the extraction of PPIm relations. While the effectiveness of entity recognition is weakened in the document triage task due to the difference in mutation characteristics, we argue that it is necessary in biocuration text mining workflow. The details are as follows.

**Table 9:** Quantitative analysis of training and test sets in terms of the number of entities (mutations or interactions identified by BioNLP tools per document. Min, Q1 (25th percentile), Median, Q3 (75th percentile) and Max give the distributional characteristics. Mean and std show the characteristics on average. The numbers marked with * show the p-value is less than 0.00001 when conducting a z-test on two samples

Entity	Class	Train vs Test	Min	Q1	Median	Q3	Max	Mean (Std)
Mutation	Relevant	Train (1729 documents)	0	0	1	2	29	1.6744 (2.6767)^*^
Test (704 documents)			0	1	2	4	24	2.6520 (3.1051)^*^
Non-relevant	Train (2353 documents)	0	0	0	1	17	0.7046 (1.6180)^*^
Test (723 documents)	0	1	1	3	20	2.2517 (2.3849)^*^
Interaction	Relevant	Train	0	7	15	22	66	15.6217 (10.9309)
Test	0	9	16	24	71	16.7528 (10.8994)
Non-relevant	Train	0	7	14	21	64	14.3366 (9.9784)
Test	0	8	15	23	55	15.7718 (11.0419)

### Error analysis and discussion of the document triage task

Through error analysis, we identify three key findings related to mutation entities. In addition, we present a small case study on the boosting logistic regression model and generalize the findings based on the observations for the submissions across all teams.

The three key findings are (i) the characteristics of mutations are significantly different between the training and test sets, specifically mutations are rarely identified in the training set but are identified in almost all the documents in the testing set; (ii) the potential utility of term lists for document triage is consistent over the training and the testing set—the distribution of mutation terms in the training set is very similar to the distribution in the testing set; and (iii) leveraging BioNLP tools and term lists is effective for the training set but not for the testing set—BioNLP tools can detect mutations for only about 50% of relevant documents in the training set but can detect more than 80% in the testing set.

#### The characteristics of mutations are statistically significant different between the training and test sets

As illustrated in Table 7, the main problem causing a dramatic performance decrease is that all the models tend to classify non-relevant documents as relevant. We thus investigate the problem by quantitatively analysing the characteristics of mutations and genes between the training and testing sets. Table 9 summarizes the number of genes and mutations identified per document by BioNLP tools.

For the training set, two observations can be made:

1. The relevant documents have an average of 1.7 (±2.7) mutations per document identified by BioNLP tools; the non-relevant documents only have 0.7 (±1.6) mutations on average, which means many non-relevant documents do not have identified mutation mentions. This difference suggests that models could use mutation-related features to differentiate the two classes.
2. The number of mutations identified in the relevant documents has a median of only 1 and many relevant documents have no identified mutations. Since all the relevant documents would be expected have at least one mutation mention, this indicates that many mutation terms cannot be identified by BioNLP tools due to being general references.

The counterpart mutation characteristics in the testing set, however, are substantially different.

1. The relevant documents have very similar number of mutations with non-relevant documents: relevant documents have 2.65 (±3.1) mutations on average while non-relevant have 2.25 (±2.4), showing that both classes have mutations identified by BioNLP tools.
2. Mutation mentions are more specific and identified by BioNLP tools. At least 75% of non-relevant documents have at least one identified mutation and most of the relevant documents in the test set have mutations identified by BioNLP tools.

We additionally perform a two-tailed z-test on the two classes (relevant documents in the training set vs relevant documents in the testing set; same for non-relevant documents) in terms of their mutation characteristics. We chose z-test for these two samples because (i) these two samples can be assumed as independent—in supervised learning, the training and the testing set should be not correlated and (ii) the sample sizes are large enough. Thus, it satisfies the prerequisites of a z-test. The null hypothesis is two-tailed: μ = μ0, meaning that the means of the two samples do not have significant differences and the alternative hypothesis is that μ ≠ μ0, meaning that the means have significant differences. We chose the confidence interval as 99.9999%. The statistical test results show that both classes have *P*-values less than 0.0001, thus rejecting the null hypothesis; we can conclude that the relevant and non-relevant documents in the training set are statistically significant different from their counterparts in the test set in terms of the mutation characteristics. Such significant difference suggests the documents in the training and testing sets are possibly from different distributions. This is supported by the descriptions of the updated dataset: the training set contains documents from both curated databases and automatic curation tools, whereas the testing set contains documents only from the automatic curation tools due to `limited annotation time’. The difference in the mutation distributions lowers the model performance and weakens the motivation to perform mutation entity recognition.

**Figure 6:**
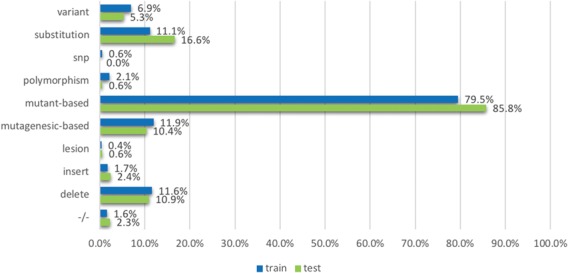
The proportion of relevant documents containing individual mutation terms, across training and testing data sets. The terms (or the groups of terms) are mentioned in the model development section.

**Figure 7:**
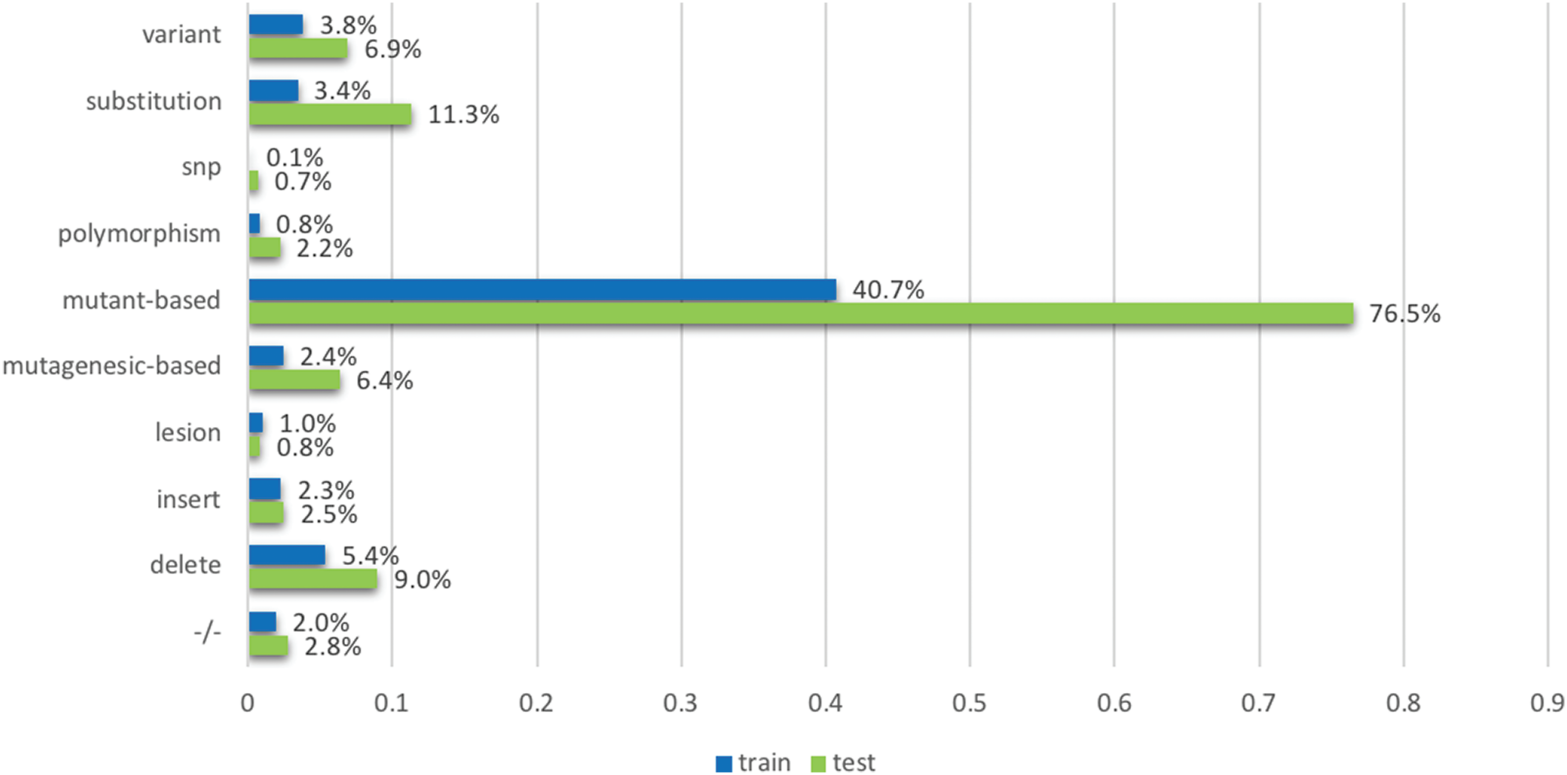
The proportion of non-relevant documents containing individual mutation terms, across training and testing data sets. The terms (or the groups of terms) are mentioned in the model development section.

**Figure 8:**
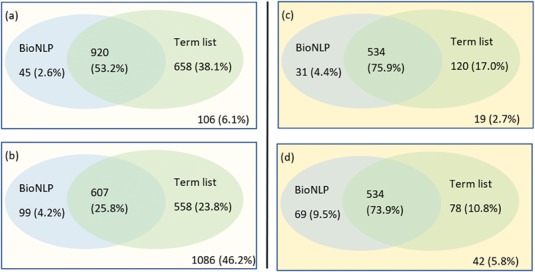
A Venn representation on number of documents have been identified having mutations using a combination of BioNLP tools and term lists. The left side shows the results on the training set: (a) for relevant documents and (b) for non-relevant documents; the right side (c) and (d) shows the results for relevant documents and non-relevant documents respectively in the testing set.

We performed the same analysis on gene-related characteristics and found that the two sets have consistent statistics; the mutation characteristics are the main point of difference.

#### The effectiveness of term lists is consistent between the training and test sets

Critically, BioNLP tools for mutation detection should have higher precision than general mutation term lists. Term lists are not intended to replace these tools, but are a complement to improve the recall of PPIm-relevant documents, as supported by the observations from the training set. Adding term lists may also add noise, for instance if a mutation term occurs frequently in the training set, but not in the testing set. We therefore also quantify the effectiveness of term lists.

Figure 6 shows the distribution of strong mutation terms contained in the relevant documents, considering the training and testing sets separately. The results show that the distribution of these terms is fairly consistent between the two sets. For example, `mutant’-based terms (mutant and mutation) are the most frequent mutation related terms in the list in the training set (79.5%), which is also true for the test set (85.8%). Likewise, the terms that are less frequent also appear in similar proportions; for instance, lesion occurred in 0.4 and 0.6% of documents in the training and testing sets and 1.7 and 2.4% for insert. Given that the distribution is fairly consistent, term lists probably do not contribute to overfitting to the training set; this is also evidenced by the feature contribution study above. In addition, the term lists (or word embeddings if using deep learning architectures) are necessary given that BioNLP tools can only achieve ~50% recall in the training set as described above. In contrast, Figure 7 shows the distribution of non-relevant documents in both sets, and demonstrates that non-relevant documents in the testing set have a much larger proportion of mutation term lists than those in the training set; for example, there are about 76% of non-relevant documents contain mutation terms whereas there are only about half of that amount in the training set. This is consistent with the previously introduced statistical analysis on mutations identified by BioNLP tools. The results collectively show that term lists are not the main factor contributing to a dramatic decrease in performance over the test set.

#### Leveraging entities is useful for the training set but less so for the testing set

We next consider the effectiveness of combining of BioNLP tools and term lists to recognize mutations and interactions. Figure 8 shows the number of documents having mutations identified by BioNLP tools only, term lists only or in combination. Figure 8(a) shows that a combination of BioNLP tools and term lists can find mutation mentions in 94% relevant documents in the training set, whereas using BioNLP tools alone can only find 55%. Figure 8(b) shows that BioNLP tools finds that 30% of non-relevant documents contain mutation mentions and a combined approach identifies 54%. We cannot quantify how many documents in the additional 24% correctly should be annotated as containing mutations, since there is no gold standard annotation available specifically for mutation mentions. However, the results indicate that the combination of BioNLP tools and term lists is effective: (i) it dramatically increases the recall as compared to using BioNLP tools only and (ii) mutations identified by the combined approach are important features to distinguish relevant and non-relevant documents (94% vs 54%).

The combined approach, nevertheless, is less important for the test set. BioNLP tools alone can already find mutations in over 80% relevant documents in that set. Comparatively, the combined approach can find mutations in 97% of relevant documents, which is consistent with the results on the training set, but does not represent as big of an improvement. Also, the combined approach identifies mutations in over 95% of non-relevant documents in the test set. This is substantially different from the training set (where only 54% of non-relevant documents had a mutation mention). This reflects our findings on the inconsistency of mutation characteristics.

#### A case study of erroneous cases in the boosting logistic regression model

We gain further insight into the data through a case study of the confusion matrix of the boosting logistic regression model. This model is interesting to study in that it achieves the best overall performance on the training set, but dramatically decreases in the test set (7% for ranked precision, 7% for F1 score and 12% for precision). We consider the four categories of evaluation: FP, a document classified as relevant but should be non-relevant; false negative, a document classified as non-relevant but should be relevant; true positive (TP), a document classified as relevant and it is correct and true negative, a document classified correctly as non-relevant. We comparatively quantitatively examine the number of mutations identified by BioNLP tools and term lists per document for each case. Tables 10 and 11 show the results for the training and testing sets. In the training set, the proportion of erroneous cases is relatively balanced: 13.5% for FP and 9.8% for FN. Looking at the FP cases, the average number of mutations identified is very close to that for TPs (for example, an average of 1.6 mutations identified for FPs vs 1.9 for TPs), leading to confusion. Conversely, FNs have many fewer mutations identified than TPs. This likely leads the model to classify such documents as non-relevant.

The pattern over the test set is very different. The proportion of erroneous cases is imbalanced; 28% for FP and 11% for FN. The number of FPs is double the number of FNs and it also is double the number of FPs over the training set (13.5%). This shows that the model built from the training set tends to classify many non-relevant documents in the testing set as relevant, thus increasing the number of FPs. Combined with the above statistical analysis on genes and mutations, we speculate that this is due to a higher proportion of non-relevant documents in the testing set have mutations, making the model infer that it is relevant. In contrast, the proportion of FN remains similar: 9.8 and 11.2%, respectively, indicating that the characteristics of relevant documents are consistent in both sets.

**Figure 9:**
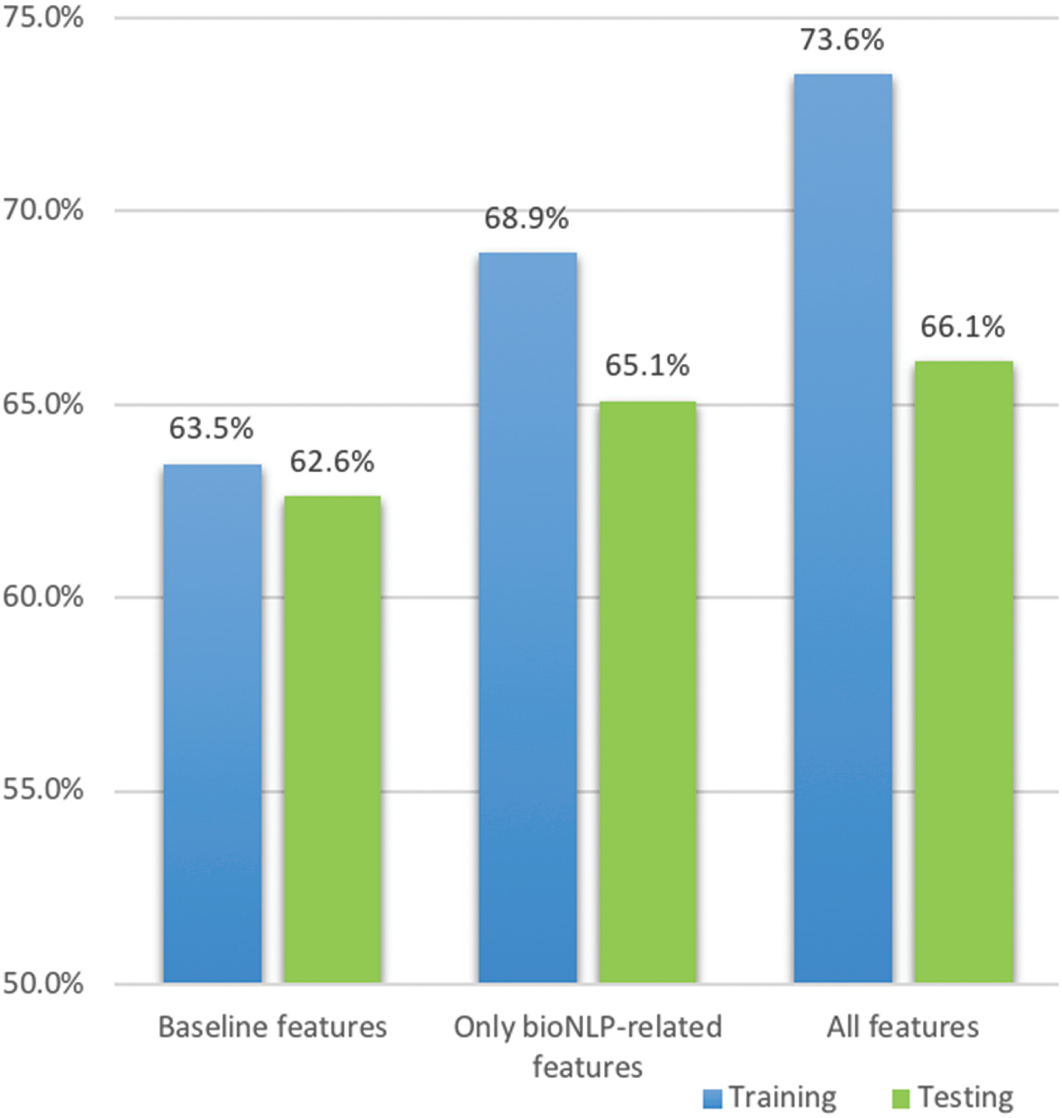
Comparative F1 scores for boosting logistic regression over the training and testing sets. The legend shows the features used in the training set and the corresponding columns are presented in the testing set.

We further compared the performance of the boosting logistic regression model using just baseline features (recall tf-idf), only BioNLP-related features (no term lists) and all the features. As illustrated in Figure 9, BioNLP tool and term list related features increase F1 score by 5% each. The 10% increase demonstrates that entity recognition is indeed effective and term lists can complement BioNLP tools in the entity recognition step. Nonetheless, the utility of entity recognition is much lower in the testing set, only increasing F1 score by 3.5% (vs 10% in the training set). More specifically, both feature sets also improve less: 2.5% for BioNLP tools and 1% for term lists.

**Table 10:** Characteristics of erroneous and correct cases classified by boosting logistic regression over the training set. Perspective: B for BioNLP tools and T for term lists. The numbers are the number of mutations identified using B or T per document

Category	# cases (%)	Perspective	Min	Q1	Median	Q3	Max	Mean	Std
FP	552 (13.52%)	B	0	0	1	2	15	1.6069	2.1651
T	0	1	2	4	16	2.8043	2.2291
FN	399 (9.77%)	B	0	0	0	1	19	0.8647	1.8803
T	0	0	1	2	13	1.3559	1.6356
TP	1330 (32.58%)	B	0	0	1	2	29	1.9173	2.8280
T	0	0	0	1	12	0.6863	1.3548
TN	1801 (44.13%)	B	0	0	0	0	17	0.4281	1.2874
T	0	0	0	1	12	0.6863	1.3548

**Table 11:** Characteristics of erroneous and correct cases classified by boosting logistic regression over the test set. The measures are the same as Table 10

Category	# cases (%)	Perspective	Min	Q1	Median	Q3	Max	Mean	Std
FP	396 (27.75%)	B	0	1	2	4	17	2.6869	2.5051
T	0	2	3	5	14	3.9444	2.9253
FN	161 (11.28%)	B	0	0	1	2	13	1.5962	2.1041
T	0	1	1	2	10	1.8758	2.0574
TP	543 (38.05%)	B	0	1	2	4	24	2.9650	3.2801
T	0	2	3	6	17	4.0166	2.8669
TN	327 (22.92%)	B	0	1	1	2	20	1.7248	2.1139
T	0	0	1	2	19	1.6575	2.0836

The case study results generalize to the results submitted by all the teams. Overall, 10 teams made 22 submissions. We trace the proposed models and results based on the workshop proceedings. Six teams apply entity recognition, either using entity recognition tools (like the tools mentioned above), entities curated by database staff, such as interactions captured in IntAct (38) and BioGrid (39), or knowledge provided by ontologies [such as interaction keywords listed in the Interaction Network Ontology (40). The top-ranked results, in terms of F1 score, are the submissions not using entity recognition. The methods used are convolutional neural network (CNN)- and recurrent neural network (RNN)-based architectures (41, 42). These methods have been shown to work well for classification tasks (43), and do not require entity recognition. Interestingly, the top-ranked team suggested adding entity recognition to the model as the future work (44). For the submissions using entity recognition, including those using CNN and RNN methods, their performance decreased substantially over the training set. One submission using a CNN model achieves over 88% F1 score via cross-validation over the training set, but has a 21% decrease over the testing set (45); another submission using CNN with RNN also has a 13% decrease (from 81 to 68%) (46). These models have applied careful regularisation, such as dropout, early stop and use cross-validation or a train/dev/test split to reduce overfitting. The large difference in the performance appears to be primarily due to entity recognition—the features derived from entity recognition are effective in the training set but not in the testing set.

Despite these results, we argue that entity recognition is critical; in the biocuration workflow entity recognition is a key early step that cannot be missed (47, 48). Even for document triage, entity recognition is still important to identify documents relevant to a specific triage task and at the same time can capture important information from those documents. Entity recognition is not effective in the specific context of the Biocreative VI Track 4 data because of the distributional differences between the training and test sets.

A further improvement of the document triage model would be to use more advanced machine learning architectures. As shown above, CNN- and RNN-based networks have been shown effective in this task. Future experiments could apply a CNN to capture important entities, as CNN models excel at object detection, together with an RNN to capture textual semantics. In the context of biocuration, entity embeddings (embeddings over genes and mutations, for example) could be more effective than raw word embeddings and may have the potential to improve the performance of neural network models. The effectiveness of such methods has been shown in relation extraction in general knowledge bases (49). Based on the existing submissions, there is no deep attempt to use entity embeddings based on state-of-art BioNLP tools to facilitate neural network models and could be a direction for future work.

Otherwise, we plan to do a thorough evaluation on different models with different feature sets to quantify the most appropriate model in document triage.

### Discussion of the relation extraction task

In this section, we present an evaluation and discussion of the relation extraction performance. In Table 8, we presented the impact of entity annotations on relation extraction. Particularly, absence of entity annotations, missed by the standard BioNLP tools, limits the recall performance of relation extraction. In this section, we study other factors in the PPIm relation extraction that impact the performance.

In this task, our goal is to extract interacting protein pairs that are affected by a mutation. Intuitively, the presence of mutation terms or entities in a sentence should help improve the performance of the task. With GNormPlus as the entity annotator for genes, we experimented with including the mutation terms lists (described in the Feature engineering section of the Document Triage task) as additional features to the classifier. This is modelled via a linear combination of kernels, namely the graph kernel and a bag of words kernel based on a bag of mutation terms. Mutation terms led to modest improvements in F-score, from 0.2877 to 0.2890 for the training set. However, including mutation terms resulted in a drop in the performance over the test set. Mutation entities are sparsely mentioned within the PPIm corpus, and as discussed previously, the distribution of these entities varies between the training and test sets; with only 1044 out of 6104 sentences in the test set annotated with mutation entities.

Another approach is to model mutation mentions as entities directly in the graph representation for the ASM kernel, via special node labels. For this approach, we use Pubtator to provide mutation mention annotations in the text. This results in a higher F-score of 0.3509 over the test set. The co-occurrence approach is not affected by the differences in the mutation terms between the training and the tests sets, simply because it does not use the mutation term features.

Adding in strategies for extracting relations across sentence boundaries (non-sentence relation extraction; discussed in Methods helps to improve the recall performance, but also produces many FPs, resulting in an overall decline in F-score. From Table 4, we observe that most (90%) PPIm relations are expressed as sentence level relations and only a small fraction of PPIm relations (10%) appear as non-sentence level relations, therefore limiting the contribution of non-sentence relation extraction. Secondly, the skewed class imbalance means that there is likely inadequate training data for the non-sentence relation classifier, hurting its generalisability.

In the co-occurrence approach, we have only extracted relationships between gene pairs that co-occur within a sentence. In order for co-occurrence approaches to take into account cross-sentential relationships, we would likely have to apply co-reference resolution (51, 52); considering document-level co-occurrence blindly would very likely result in very high number of FPs. Co-reference resolution would enable identifying indirect references to entities; these references could be substituted with their referent. For instance, phrases like `the protein’ would be replaced with the actual name of the protein itself. This means that once the co-reference expressions are resolved, we can work with sentence level relationships.

We experimented with including the document relevance score from the document triage as a feature provided to the graph-based relation extraction system, with the hypothesis that documents with a higher relevance score are more likely to contain PPIm or TP relations. This feature does improve the precision slightly (0.3384 to 0.3467) but also results in a reduced recall (0.3567 to 0.3383) and an overall reduction in F-score for the test set.

In summary, the main performance bottleneck for PPIm relation extraction task is in entity recognition. Improving the recall performance of entity recognition for genes and mutations is likely to be a prerequisite step before further improvement in relation extraction performance can be obtained. Our machine learning approach is also narrowly focused on individual sentence-level analysis, in contrast to the co-occurrence approach that tries to aggregate the evidence for a protein pair across all mention pairs in the document. Extending the machine learning approach for document level relation extraction is likely to improve its performance further.

The advantage of the co-occurrence approach that we tested is its simplicity and intuitiveness. Its relative success in the context of the relation extraction task can be attributed substantially to the fact that it leverages characteristics of the task setup, specifically the fact the documents/abstracts are known to contain at least one PPIm relationship (i.e. it begins with articles that are positive outputs of document triage). So the problem then reduces to filtering the invalid relationships out of the pool of protein co-occurrences, that is, in principle the approach begins with perfect recall and must recognize and remove only FPs.

For this challenge, this simple method worked very well. With an entity oracle, i.e. given perfect annotation of the protein entities in the test set, we could have achieved an F1-score of ~79% with N ≥ 3. Increasing the threshold to N ≥ 9 increases the theoretical F1-Score to ~95% for the test set using all the gold annotated entities. This theoretical high score in the test, without a drop in the recall despite increasing *N* as seen in Figure 5, is because of the default rule H3, that comes into effect for protein pairs that fall below the threshold. The lower maximum achievable F-score in the oracle condition in the training set as compared to the test set is due to the third (default, H3) rule applying in fewer cases in that data sample. This is another example of a substantive difference in the characteristics of the training and test sets.

## Conclusions

The PPIm dataset for BioCreative VI Precision Medicine Track supports the development of automated natural language processing tools to support the discovery of protein interactions influenced by mutations, a task of substantial importance in precision medicine applications. Our efforts in the context of the shared task show that differences in the distribution of mutation mentions between the training and test sets limit the generalisability of trained models and the reliability of performance evaluation. For relation extraction, we also found that machine learning models were outperformed by a carefully designed co-occurrence approach. While there is room for improvement, there is also clear evidence that automated methods have good potential to tackle the open challenges in both document triage and relation extraction in this context.

In the future, we plan to explore the application of deep learning architectures for the document triage task to better capture document semantics, to improve differentiation of relevant and non-relevant documents. For relation extraction, we plan to use joint models for entity recognition and relation extraction to address the limitations of the entity recognition phase in this task; this approach has shown promise in recent work (52). There are opportunities to develop newer models that combine the two approaches of machine learning and co-occurrence, to exploit both linguistic clues and task-specific heuristics to achieve higher performance.

## Notes

*Availability*: The code is available from https://bitbucket.org/readbiomed/biocreative-vi.
